# Extraction of canine gait characteristics using a mobile gait analysis system based on inertial measurement units

**DOI:** 10.1016/j.vas.2023.100301

**Published:** 2023-06-10

**Authors:** M. Altermatt, D. Kalt, P. Blättler, E. Schkommodau

**Affiliations:** a Institute for Medical Engineering and Medical Informatics, School of Life Sciences, University of Applied Sciences Northwestern Switzerland, Hofackerstrasse 30, Muttenz CH 4132, Switzerland; b Orthovet, Fasanenstrasse 13, CH 4402 Frenkendorf, Switzerland

**Keywords:** Inertial measurement unit, Canine gait, Movement analysis, Gait characteristics, Range of motion

## Abstract

This study aims to investigate two simple algorithms for extracting gait features from an inertial measurement unit (IMU) based canine gait analysis system. The first algorithm was developed to determine the hip/shoulder extension/flexion range of motion. The second algorithm automatically determines the stance and swing phase per leg. To investigate the accuracy of the algorithms, two dogs were walked on a treadmill and measured simultaneously with an IMU system, an optical tracking system and two cameras. The range of motion estimation was compared to the optical tracking systems, with a total of 280 steps recorded. To test the stance and swing phase detection, a total of 63 steps were manually annotated in the video recordings and compared with the output of the algorithm.

The IMU's-based estimation of the range of motion showed an average deviation of 1.4° to 5.6° from the optical reference, while the average deviation in the detection of the beginning and end of the stance and swing phases ranged from -0.01 to 0.09 s. This study shows that even simple algorithms can extract relevant information from inertial measurements that are comparable to results from more complex approaches. However, additional studies including a wider subject pool need to be conducted to investigate the significance of the presented findings.

## Highlights

• Simple algorithms could extract gait characteristics based on inertial measurement units (IMU).
• The comparison of the IMU-based estimation of the range of motion with the optical tracking system gave similar results to the more sophisticated IMU-based approaches.
• The IMU-based detection of stance and swing phases compared to video-recorded steps showed promising results.

## Introduction

Objective gait analysis can provide clinicians with important information for therapeutic decision-making. It can be used not only to quantify and differentiate gait for diagnosis, but also to monitor rehabilitation and treatment efficacy (Sandberg et al., 2020). In addition, objectively collected data can provide important information for breeding decisions.

Gait analysis systems currently used in veterinary medicine to collect kinematic and kinetic data are either camera-based systems (Foss et al., 2013), force plate systems (Duerr, 2020), accelerometer-based systems (Ladha et al., 2017), surface electromyography measurement systems (Deban et al., 2012) or instrumented treadmills (Gillette & Angle, 2008; Volstad et al., 2016). Camera-based systems that track optical, active, or passive markers (Foss et al., 2013) attached to the dog's body are commonly used in research facilities (Fu et al., 2010) but rarely in veterinary clinics because they are very expensive (Gillette & Angle, 2008) and require a dedicated space to set up the system. Ground reaction force measurement systems, such as force plates, have been shown to be accurate indicators of irregular gait patterns or lameness, especially when combined with camera-based motion tracking devices, but require a long acclimatization period and training of the dog to the walking surface (Fanchon & Grandjean, 2007).

All of the above systems have limitations for daily use in veterinary practice, either because a special room for measurement or acclimatization of the dog (Clark et al., 2014; Lopez et al., 2006) is required, or repetition of measurements is necessary to capture the whole-body movement during gait (Barthélémy et al., 2009). In addition, the capital cost of the mentioned systems, as well as the time required to set up the measurement is very high. Several studies indicate that inertial measurement unit systems provide valuable information for the analysis of the canine gait (Duerr et al., 2016; Gillette & Angle, 2008; Lane et al., 2015; Lopez et al., 2006). In a study by Clark et al. (Clark et al., 2014) the peak vertical forces (PVF) measured with a force platform were compared with measurements from a triaxial accelerometer placed dorsally over the thoracic or lumbar region. There was positive and significant agreement between the PVF of the accelerometer and the force platform for the forelimbs and positive and low agreement for the hindlimbs. Barthélémy et al. (Barthélémy et al., 2009) described the use and reliability of accelerometers in gait assessment of healthy dogs and dogs with a diagnosis of muscular dystrophy. Duerr et al. (Duerr et al., 2016) reported that kinematics recorded with an inertial measurement unit (IMU) in the sagittal plane in dogs, showed good correlation with optically recorded kinematics, so the use of IMU sensors could provide an alternative to optical kinematic gait analysis while allowing data collection outside the laboratory. Ladha et al. (Ladha et al., 2017) presented an IMU sensor-based gait measurement system for dogs that demonstrated good sensitivity and repeatability with a precision likely sufficient to detect clinically relevant gait abnormalities in dogs. They concluded that, with further development, the system could have a wide range of applications in both research and clinical practice. Recently, Zhang et al. (Zhang et al., 2022) also achieved promising results with a very similar approach. Comparable IMU systems (IMUS) are used in human medicine and, to some extent, in equine practice for diagnosis, rehabilitation, and research on the efficacy of interventions. The results of the present study suggest that similar benefits could be achieved if the evaluated detection methods were developed into an end-to-end solution for veterinary practice in dogs (Ladha et al., 2017).

The aim of our work is the evaluation of simple IMUS-based approach for estimating the range of motion and stance/swing phases in the canine gait.

## Materials and methods

**Fig. 1:**
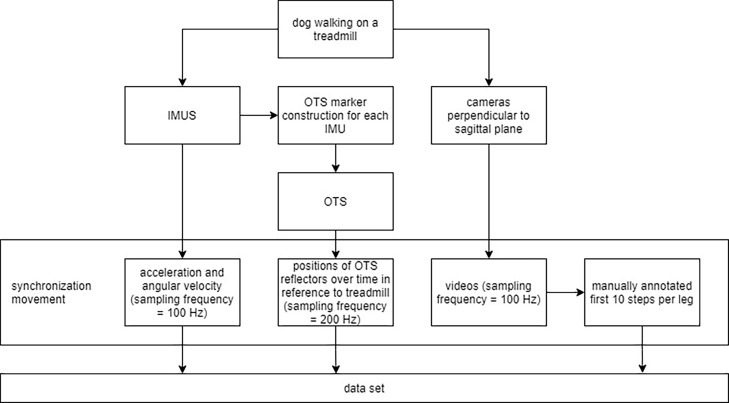
Data acquisition and system synchronization: During the measurements, three separate systems were used simultaneously to acquire information. A synchronization movement enabled synchronization between the different measurement systems. As a result, the data set consists of the acceleration and angular velocity signals measured by the IMU system, the time-dependent positions of the Optical Tracking System (OTS) reflectors, and the manual annotation of the gait cycles of ten steps per leg and measurement using video cameras.

To investigate the possibility of extracting range of motion of hip/shoulder extension/flexion and stance and swing phase based on inertial measurement units, dogs were simultaneously measured with an inertial measurement unit system (IMUS), optical tracking system (OTS) and optical cameras while walking at constant speed on a treadmill (Fig. 1).

Data were collected from two dogs, a *Labrador retriever*, male, age 7 years, and an *Altdeutscher Tiger*, male, age 11 years. In this document, they are referred to as "dog A" and "dog B." There were no known pathologies in either dog, which was confirmed by a certified clinician prior to the measurements. The measurements were conducted in context of research with dogs (animal experimentation permit (Form B) BL499 29,080; animal experimentation application BL499 29,080; national ethical standards in Switzerland).

### Setup of IMU system

**Fig. 2:**
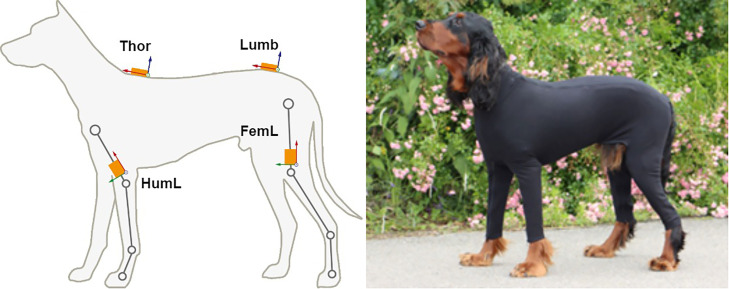
Left: sensor positions and coordinate systems; Right: Sensor fixation vest.

An IMU gait analysis system (LupoGait, 4Dvets, Interlaken, Switzerland) consisting of six IMU sensors (MTw Awinda, Xsens Technologies B.V., Enschede, The Netherlands) attached to the dog's body with a special sensor fixation vest was used for the study (Fig. 2). Of the six sensors, two are attached to the humeri (sensor positions *HumL* - humerus left - and *HumR* - humerus right), with the x-axes of the sensors aligned with the humerus and pointing upward. The z-axes of the sensors are aligned perpendicular to the sagittal plane and point away from it. Two additional sensors are analogously attached to the femora (sensor positions *FemL* - left femur - and *FemR* - right femur). In addition to the four leg sensors, another pair of sensors is attached to the spine. The first sensor is located on the thoracic segment (sensor position *Thor*) and the second on the lumbar segment of the spine (sensor position *Lumb*). The orientations of the spine sensors are identical. The z-axis points upward and the x-axis is aligned with the spine and points in the direction of travel. The IMU sensors provide synchronized acceleration, angular velocity and magnetometer data acquired at a sampling rate of 100 Hz.

### Setup of optical tracking system

**Fig. 3:**
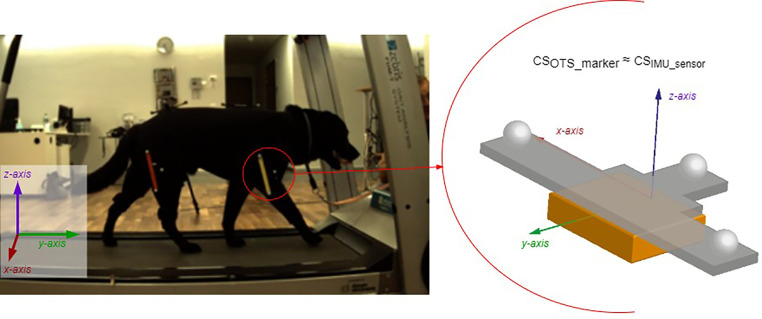
Left: Measurement set up: Dog with optical markers and IMUS on treadmill. The arrows indicate placement and orientation of the reference system for the OTS; Right: OTS marker on IMU sensor: The OTS marker coordinate system and IMU sensor coordinate system are identical.

Besides using an IMUS, the dog's walk was recorded on a treadmill using an optical tracking (OTS) system (Vantage (six cameras), Vicon Motion Systems Ltd UK) and two optical cameras (Bonita 720C, Vicon Motion Systems Ltd UK), positioned left and right perpendicular to the sagittal plane of the dog (Fig. 3, left). The OTS markers were fixed with defined orientation in relation to the IMU sensor to allow definition of OTS marker coordinate systems aligned with the corresponding IMU sensor coordinate systems during the data processing (Fig. 3, right). The OTS origin of coordinates was defined to be the left corner of the treadmill behind the dog (Fig. 3, left).

### Recording procedure

At least three measurements per dog of one minute duration were performed. After a brief familiarization period (approximately 20 to 30 s) on the treadmill, the dogs were walked at a speed between 3.5 and 4.0 km/h. The speed of the treadmill was adjusted so that the dogs remained walking during the measurements.

Data collected for the IMUS included linear acceleration and angular velocity at a sampling rate of 100 Hz. With the OTS, data were collected from each OTS marker with respect to the global OTS coordinate system attached to the treadmill at a rate of 200 Hz. The sampling rate (frame rate) of the video cameras used was 100 Hz. To enable synchronization of data from all three measurement systems, a special routine (rapid, jerky movement of an OTS marker attached to an IMU sensor) was used at the beginning of each measurement.

### Extracting joint angles

In optical gait analysis, flexion and extension angles as well as range of motion (ROM) can be calculated based on the measured marker positions (Foss et al., 2013). In IMUS, the sensor positions cannot be calculated exactly because the starting positions for the required integration are not known without error accumulation. Nevertheless, in the next section we show an approach for extracting joint angles.

#### IMUS based joint angles

The z-axis of the IMU sensor of the leg sensors represents approximately the main axis of rotation of the leg in the sagittal plane during regular gait. The hip or shoulder flexion and extension angles were estimated by cumulative integration of the z-signals of the gyroscope of each leg sensor. To normalize the signals, the z-axis direction of the sensor for the left side was mirrored to the sagittal plane. The offset and drift underlying this integrated signal were filtered with a high-pass filter (Chebyshev 3rd order, 1.2 Hz cut-off frequency) to obtain an angle estimation signal for each leg. More sophisticated angle estimation algorithms, similar to works of Seel et al. (Seel et al., 2014) were tested. However, those approaches were not considered robust enough for the application at hand.

#### OTS based joint angles

To calculate ROM from optical tracking, the angle of the OTS marker between the x-axes of the leg sensors and the x-axes of the corresponding back sensors (e.g., the angle between the x-axis of the left humerus and the x-axis of the thoracic sensor) was calculated over time.

#### Evaluation

Synchronization between the OTS and IMUS angle signals was fine-tuned by eliminating phase shifts using cross-correlation. The OTS and IMUS angle signals were divided into segments based on the positive peaks in the IMUS angle signals. This segmentation corresponds to a subdivision into step cycles per leg. From each OTS signal segment, the offset of the segment itself was subtracted to eliminate the offset between the IMUS angle estimates and the OTS reference angles.

Sequences with 40 steps per leg and measurement were selected for analysis from the synchronized angle signals. The analysis includes the calculation of the IMUS- and OTS-based ROM, the difference between them, and the root-mean-square-error (RMSE) between the IMUS- and OTS-based angle signals. All four metrics were calculated per step before being combined into an average value and standard deviation across all measurements per leg and dog. In addition, the correlation between the IMUS and OTS-based angle signals was calculated for all steps per leg position and dog.

### Stance and swing phase determination

An IMUS-based method for identifying the swing and stance phase was to be evaluated. For this purpose, the approach was compared with manual video annotation.

To detect stance and swing phases with an IMUS, minima and maxima were determined in the cumulative integrated gyroscope z-axis signal of each leg sensor. The signals from the right side (right femur/humerus) were mirrored on the sagittal plane to achieve normalization for maxima/minima detection. The minima peaks in each cumulative integrated gyroscope sensor z-axis signal should de-scribe the initial contact points (IC points) where the swing phases end and the stance phases begin. The maxima peaks describe the final contact points (FC points) where the corresponding paw has left the ground and the swing phase begins.

The reference consists of nine annotated IC-/FC-points per leg per measurement which were compared to the corresponding detection. To evaluate the time differences, the absolute time differences relative to the step duration based on the annotation were calculated for each IC-/FC-point. The differences were averaged for each dog's leg. The average values for dog A are based on 27 steps (three measurements, each with nine video-annotated steps). For dog B the values are derived from 36 steps. Additionally, the standard deviations were calculated.

## Results

### Joint angles

Comparing inertial measurements-based angle signals to the ones based on optical measurements results in mean RMSE per step in the range of 1.8° up to 3.9° and Pearson's correlation coefficients between 0.94 and 0.98 (Table 1). Table 2 describes the ROM of the two systems and the difference between them. The error of the inertial measurement system to the optical system reaches mean values of 5.6° The ROM errors for dog A are higher than for dog B. This is true for all sensor positions. In addition to the difference in error, the ROM values of dog A show an imbalance between the femora. The forelegs of both dogs also show asymmetries, although to a lesser extent.

**Table 1:** Results of comparison between time-dependent joint angles of all steps per leg (including 120 gait cycles for dog A and 160 for dog B). The middle column describes the Pearson's correlation between IMUS and OTS angle signal. The right column lists the average root-mean-square errors per gait cycle.

Sensor position	Subject	correlation of joint angle over time between OTS and IMUS	RMSE per Step [°] mean ± SD	
HumL	dog A	0.98	2.0	± 0.46
	dog B	0.96	2.5	± 0.35
HumR	dog A	0.98	1.8	± 0.64
	dog B	0.95	2.1	± 0.61
FemL	dog A	0.94	3.9	± 0.50
	dog B	0.95	2.9	± 0.40
FemR	dog A	0.96	2.6	± 0.37
	dog B	0.96	2.5	± 0.38

**Table 2:** Results for range of motion of hip/shoulder flexion/extension based on IMUS or OTS averaged over all gait cycles of the same dog (included: 120 gait cycles for dog A and 160 for dog B).

Sensor Position	Subject	ROM IMUS [°]		ROM OTS [°]		Error ROM [°]	
		mean	± SD	mean	± SD	mean	± SD
HumL	dog A	25	± 1.5	28	± 1.6	3.4	± 1.51
	dog B	22	± 1.2	26	± 1.2	2.6	± 1.27
HumR	dog A	24	± 1.8	26	± 1.4	2.5	± 1.25
	dog B	21	± 1.1	23	± 1.3	1.4	± 1.07
FemL	dog A	34	± 2.2	37	± 2.7	4.4	± 2.41
	dog B	26	± 1.3	31	± 1.6	2.6	± 1.42
FemR	dog A	28	± 1.5	33	± 1.7	5.6	± 1.93
	dog B	27	± 1.3	29	± 1.6	1.7	± 1.24

### Swing and stance phases

Table 3 shows the time differences between the IMUS determined initial and final contact points and the video labeling. The differences are in the range of −0.01–0.04 s for the detection of the initial contact and values of 0.01–0.06 s for the detection of the final contact point. Since both IC and FC detection show predominantly positive differences, the duration errors of the stance and swing phases are even lower. In relation to the annotated step duration, the difference of IC and FC detection to video labeling is between 2 and 9%.

**Table 3:** Average time differences and corresponding standard deviation between initial and final contact points per leg of each dog. The study includes 27 steps per leg for dog A and 36 steps for dog B. In addition to the time differences, the average absolute differences between initial and final contact points were calculated in relation to the step duration derived from the annotation.

Sensor Position	Subject	Initial Contact Point				Final Contact Point			
		difference [s]		rel. difference [-]		difference [s]		rel. difference [-]	
		mean ± SD		mean ± SD		mean ± SD		mean ± SD	
HumL	dog A	0.03	± 0.01	0.05	± 0.02	0.01	± 0.01	0.02	± 0.01
	dog B	0.04	± 0.01	0.05	± 0.01	0.02	± 0.02	0.03	± 0.02
HumR	dog A	0.01	± 0.01	0.02	± 0.02	0.01	± 0.01	0.02	± 0.02
	dog B	0.03	± 0.01	0.04	± 0.01	0.02	± 0.01	0.03	± 0.01
FemL	dog A	−0.01	± 0.01	0.02	± 0.01	0.04	± 0.02	0.06	± 0.02
	dog B	0.02	± 0.01	0.03	± 0.01	0.06	± 0.01	0.09	± 0.02
FemR	dog A	0.00	± 0.01	0.02	± 0.01	0.01	± 0.01	0.02	± 0.02
	dog B	0.03	± 0.01	0.04	± 0.01	0.05	± 0.01	0.07	± 0.01

## Discussion

The angle estimation based on the IMUS with mean RMSE per step in the range of 1.8° to 3.9° to the optical reference is comparable to the result of Duerr et al. who conducted a similar investigation (Duerr et al., 2016). The IMUS algorithm generally underestimates the peak angle values. This could be due to the IMU's built-in filtering, which smooths the peak angular velocity values at a given movement speed and sampling frequency. Another reason could be a slight deviation in the orientation of the sensor's z-axis. Such deviations lead to a shift of the angular velocity to other axes during extension and flexion. Therefore, using an integrated angular velocity signal around the z-axis for angle estimation results in a lower ROM estimate.

The results of stance and swing phase detection show small temporal differences between IMU-based detection and video annotation. The differences are near the limits of the sampling frequency. The reasons for the observed offset may be a discrepancy between the selected target signal attributes (peaks in the z-axis cumulative integrated gyroscope signal) and the actual ground contact events. The step detection algorithm basically identifies the peaks in the IMUS angle signal as initial or final contact points. The step information obtained from the inertial measurements is very indirect because there are two joints between the sensors and the ground. Both joints dampen the paw impacts and obscure corresponding signal characteristics. Exploiting the peaks in the angular signal is the most robust method we found for step detection with this sensor setup. Another reason for the temporal offset could be the annotation itself. The manual annotation of step events could possibly cause a systematic time shift. A more objective method would be to use a treadmill with pressure sensors as Zhang et al. did (Zhang et al., 2022).

Overall, the present study shows that the extraction of relevant gait features is possible with inertial measurement devices and simple algorithms. Although the results for both algorithms are promising from a technical point of view, their accuracy could vary for a wider subject pool. Important factors causing possible variation could be breed, size, weight, body shape, pathology and gait pattern. Several additional studies need to be conducted to investigate such factors.

## Conclusions

This study shows that even simple algorithms can extract relevant gait features such as range of motion, stance phase and swing phase from inertial measurements. The results are comparable to other, more sophisticated approaches. However, for diagnostics, the presented approaches need further studies with a larger number of subjects, different gait patterns and pathologies. Furthermore, the reproducibility of sensor placement needs to be validated.

## Ethical statement

This manuscript matches the principles of the Committee on Publication Ethics. Furthermore, the involved use of animal subjects is according with the NC3Rs ARRIVE Gudelines.

## Declaration of Competing Interest

Two co-authors are respectively co-founder and shareholder of 4DVets AG, the manufacturer of the discussed system. All authors worked together on a research project financed by Swiss Funding Agency Innosuisse.
